# NEWS

**DOI:** 10.7189/jogh.02.020201

**Published:** 2012-12

## AFRICA

• Claims that the interventions of the Millennium Villages Project had resulted in a triple reduction in child mortality rates, as declared in a paper published in May, were followed by harsh criticism. Critics, including Michael Clemens from the Center for Global Development in Washington, called the paper’s quantitative evidence into question. In a letter to *The Lancet*, Clemens argued that the project needed greater transparency, including independent analysis. The criticism was accepted and the paper was partially retracted in a subsequent edition. Furthermore, the project’s founder, economist Jeffrey Sachs, agreed that the project was too autonomous and has now established an external advisory board to oversee the analysis of the project’s impact. This move should increase transparency and improve cost–effectiveness analysis of the project, with both factors being essential to development policy. (*Nature*, 12 Jun 2012)

• A meeting was held in Kampala earlier this year, addressing the so–called ‘nodding disease’ or ‘nodding syndrome. This illness has affected more than 3000 people in the north of Uganda, with symptoms including involuntary nodding, neurological deterioration and, in many cases, death. Historically, ‘nodding syndrome’ was already reported in the 1960s in parts of Tanzania and in South Sudan in the 1990s. In this latest outbreak in 2011, hundreds of cases were reported in northern Uganda, a region emerging from a decades–long conflict with the Lord’s Resistance Army. (*IRIN*, 2 Aug 2012)

• Oxfam has warned that the east of the Democratic Republic of Congo faces a catastrophic humanitarian crisis. In this region, millions of people found themselves at the mercy of militias, with a sharp increase in killings, rapes and looting. They also warned of a risk of cholera epidemic, with insecure environment preventing aid delivery. (*BBC*, 7 Aug 2012)

• A campaign to protect 50 million people against meningitis through vaccination has been launched in seven African countries of the so–called ‘Meningitis Belt’ – Benin, Cameroon, Chad, Ghana, Nigeria, Senegal and Sudan. Mr Seth Berkley, managing director of GAVI Alliance, is hoping for “a dramatic impact across the continent”. (*AFP*, 4 Oct 2012)

• The US pharmaceutical company Johnson & Johnson has allowed generic manufacturers to produce cheap versions of its HIV/AIDS drug ‘Prezista’ for sale in Sub–Saharan Africa and least–developed Countries. Prezista, known in its generic form as ‘darunavir’, is a new antiretroviral drug that is used when patients develop tolerance for and become resistant to existing antiretroviral treatments. The need for such drugs is growing, as increasing numbers of patients become refractory to current treatment, especially in Africa. International pharmaceutical companies are under increasing pressure to make medicines more affordable to poorer nations. (*Reuters*, 29 Nov 2012)

## ASIA

•Hand, foot and mouth disease (HFMD) has been identified as the likely cause of death of 54 children in southern Cambodia between April and July 2012. HFMD is caused by enteroviruses, most commonly coxsackievirus A16, which normally results in a mild and self–limiting disease. The Cambodian outbreak was reportedly caused by enterovirus 71 (EV–71) that causes meningitis and encephalitis, resulting in severe neurological, cardiovascular and respiratory problems. (*Reuters*, 8 Jul 2012)

• The Pakistani government announced plans to introduce a programme of pneumococcal immunisation in the country as part of their expanded programme on immunisation (EPI). The programme will be introduced in Punjab province and will be expanded nationwide within the next eighteen months. Pakistan is the first south Asian country to announce plans to vaccinate against pneumococcus and the project is mainly being funded by the GAVI Alliance. Pakistan is currently paying 20 cents of the full price of US$ 3.50 per dose, and the vaccination will be free to the user. The vaccination used will be GlaxoSmithKline’s Synflorix, which has been made available through the GAVI Alliance’s Advance Market Commitment (AMC) programme. AMC provides incentives for companies to offer products at reduced prices in exchange for large and long–term contract promises from countries. (*The Guardian*, 9 Oct 2012)

• More than 500 000 children across Myanmar will be protected against five major childhood diseases in the next six a months. The country's move to introduce the five–in–one pentavalent vaccine is a result of international partnership and support from the GAVI Alliance. (*Health Canal*, 6 Nov 2012)

• According to the Union of Syrian Medical Relief Organizations (UOSSM), it appears that Syrian troops are seizing foreign aid and reselling it, or otherwise providing it to government loyalists. In a country that is gripped in internal conflicts, this is putting millions of lives at risk. (*Reuters*, 7 Nov 2012)

• At the conference in Doha, Qatar, UN Climate Change Conference was held in December. One of the main ideas proposed at the conference was to establish the United Nations–led partnership for innovative models for financing climate change adaptation and mitigation activities. The initiative would complement the UN Framework Convention on Climate Change (UNFCCC) and the activities of the World Economic Forum (WEF), aiming to promote successful public–private financing mechanisms. (*UN News*, 27 Nov 2012)

## AUSTRALIA AND WESTERN PACIFIC

• Collectively, the adult population of the world (18 years and more) weighs about 316 million tons, according to a study published in the journal BMC Public Health to coincide with Rio +20 meeting. As 16.5 million tons of that weight is due to overweight, this amounts to 242 million people of normal weight in addition to the current global population – or roughly, the entire population of Indonesia. The authors of the study suggest that overweight pandemic could endanger both the world’s food security and environmental resources. (*Time*, 18 Jun 2012)

• The controversial Trans–Pacific Partnership Agreement (TPP) has recently come under increased public scrutiny. There are concerns that this effort, proposing to use free trade to impose conditions and override domestic laws, has little to do with trade, because actual trade barriers between the countries in the region are already very low. Some proposed provisions, such as stronger copyright and patent protection, would in fact prevent free trade. (*The Guardian*, 27 Aug 2012)

• AusAID funds research that should improve the effectiveness of Australian aid in developing countries. Between July 2007 and June 2012, AusAID provided more than 100 million AUD in support of research examining how to improve health systems and improve delivery of health–related aid in low resource settings. AusAID has now developed a longer–term strategy that will support medical research aiming to remove the barriers to achieving priority health outcomes in the Asia Pacific. AusAID’s focus is on the diseases and health issues that have the largest impact on the lives of poor people, with the main focus on the Asia–Pacific region. (*AusAID*, 10 Sep 2012)

• The founding partners of the Measles & Rubella Initiative have recently reported that measles cases are at an historic low in the Western Pacific Region, which is making excellent progress towards eliminating the measles virus. Only two measles deaths were reported this year, representing a 99% decline since 2003. (*UN Foundation*, 13 Sep 2012)

• World Health Organization hailed several success stories in the Western Pacific region that evolved during the past decade. In 2003, a plan was laid out to eliminate measles and control hepatitis B, and to use those activities to strengthen routine immunization services. Now, hepatitis B infection rates in children are reduced to below 2%, while measles deaths are nearly eliminated. The measles initiative is now also used to improve the prevention of congenital rubella syndrome. (*WHO*, 24 Sept 2012)

## CHINA

• Successful use of compulsory licenses to terminate the monopoly on expensive drugs from some pharmaceutical companies, which began in India, has now prompted changes across Asia. China has amended its patent law to allow issuing of compulsory licenses to the domestic qualified companies to produce and export generic patented drugs under certain conditions. As similar precedents had been made in other countries, China was well prepared on legal grounds for this change. It should allow the government to realize the long–term interest in producing new drugs, particularly those required to address a surging number of HIV cases. (*Reuters*, 8 Jun 2012)

• Ten years ago, one in twenty pre–school children in China was infected with hepatitis B, which was one of the highest infection rates worldwide. Today, this figure has decreased to less than 1%, in line with most industrial countries, which is a remarkable public health achievement. China’s reduction in hepatitis B infection rates will precipitate a marked decline in liver disease in adulthood. Moving forward, the country has set itself another bold task: to reduce the prevalence of hepatitis B amongst the adults to 2%, down from the current 7.2%. (*China Daily*, 13 Jun 2012)

• Chinese authorities have arrested nearly 2000 people as part of a nationwide crackdown on counterfeit drugs and health care products. There are growing concerns about the true prevalence of both fake drugs and unsafe food supplies in China, with counterfeiting operations becoming increasingly sophisticated. (*New York Times*, 5 Aug 2012)

• At this summer’s fifth Conference of the Forum on Africa–China Cooperation, the Chinese government pledged US$ 20 billion of new aid to Africa, which signals China's increasingly leading role in international development aid. (*The Washington Times*, 14 Aug 2012)

• At the first high–level meeting between the Health Minister of China, Professor Chen Zhu, and GAVI Alliance representatives, the Chinese Government expressed willingness to expand its collaboration with the Alliance. (*GAVI Alliance*, 12 Sept 2012)

## EUROPE

• The World Bank has recently advised developing nations to invest in long–term, sustainable infrastructure programmes that would be relatively independent from the volatility of the global markets. This economic anxiety has been the main reason behind reduced foreign investment into developing nations, and has seen loans from European banks decline by 40% compared with only a year ago. International financing is an essential contributor to growth and development in many of these countries. Governments of developing nations are being urged to adopt policy allowing sufficient flexibility to sustain economic growth even if global markets are to collapse again. (*The Guardian*, 12 Jun 2012)

• The Anti–Counterfeiting Trade Agreement (ACTA), whose aim was to standardize intellectual property protection, was rejected by the European Parliament earlier this year. This was the first instance that the European Parliament has exercised its powers under the Lisbon Treaty to reject an international trade agreement, with only 39 votes in favour and 478 opposing. The treaty was criticized as being vague, with the potential to jeopardize civil liberties. Before the ruling, European Parliament saw public demonstrations and received a petition from 2.8 million citizens worldwide urging it to reject the ACTA. In a detailed analysis, IP–Watch quoted Aziz–ur–Rehman of MSF saying that “...the way it was written, ACTA would have given an unfair advantage to patented medicines, and restricted access to affordable generic medicines to the detriment of patients and treatment providers alike.” (*The Guardian*, 4 Jul 2012)

• Ministers and State Secretaries of Europe came together for a meeting in Cyprus in late August to discuss the next 7–year budget for the European Union (2014–2020). Up to € 51 billion was proposed for development assistance for the world’s poorest, but this proposal is now under threat among many others, as several governments want to reduce the overall EU budget given the economic climate. (*ONE International*, 29 Aug 2012)

• As a part of its activities to aid Africa, Russia decided to write off US$ 20 billion of debts owed by African countries and to donate further US$ 50 million to the poorest countries. It is also taking part in peacekeeping operations in Africa and expanding programmes to train African peacekeepers and law enforcers, according to Mr Vladimir Sergeyev, director of the Russian Foreign Ministry Department of International Organisations. (*AFP*, 18 Oct 2012)

• Several campaigns are trying to prevent cuts to EU's proposed aid programmes for some of the world's poorest countries. UK is among the countries calling for cuts in the EU's 2014–2020 € 1 trillion budget, but campaigners fear that the aid budget will take a disproportionate hit of about 11%, which would be the highest cut in the EU budget. The group ONE released a report that suggested that EU aid would more than pay for itself by 2020 and therefore does not deserve to be seen as low priority investment. There are still hopes that a financial transactions tax could be pushed by France and other EU countries, which could increase the aid funding. (*The Guardian*, 20 Nov 2012)

## INDIA

• India will fail to meet Millennium Development Goal targets for both child and maternal health, according to the World Health Organisation's country representative, Dr Nata Menabde. Despite significant improvements, India is predicted to record under–five mortality rate of 52 per 1000 live births in 2015, which would fall short of the 42 per 1000 target. WHO added that achieving universal measles immunisation for infants should be a priority, and India is planning to reach an 89% immunisation rate by 2015. UNICEF also suggested that increased attention should be given to neonatal deaths, where cost–effective interventions are available, such as promotion of exclusive breastfeeding and clean cord care through early postnatal visits. (*Times of India*, 2 Jul 2012)

• The Indian government has quietly adopted a US$ 5.4 billion policy that will provide free medication to the population. Doctors in India’s public health service will soon be allowed to prescribe generic drugs to patients free of charge. This will see a dramatic improvement in the people’s access to health care, changing the lives of many millions, and helping to overhaul a system that made health care a luxury. The limitation to generic–only drugs should benefit Indian producers of generics, whilst large pharmaceutical companies such as Pfizer, GlaxoSmithKline and Merck will lose out. Following completion of a two–year rollout, the policy is anticipated to provide 52% of the population with free drugs by 2017. Providing free medicine is just one among many current strategies to improve health care in India. (*Reuters*, 5 Jul 2012)

• The biotech sector in India is becoming a vibrant and booming market. Its vaccines sector witnessed a continued growth in 2011–2012, with Haffkine Biopharmaceutical and Serum Institute recording growth of 100%and 60%, respectively. However, not all stories are of a success, as Bharat Biotech, Panacea Biotec, and Shantha Biotechnics saw some of their products losing the WHO pre–qualification status. Most of these companies are expected to recover as they apply corrective measures. (*BioSpectrum Asia*, 7 Aug 2012)

• India plans to ban 91 drugs from over–the–counter sale to prevent their widespread misuse. These include 73 antibiotics, 13 drugs that are potentially addictive, and four anti–tuberculosis treatments. New provisions should ensure that these drugs can only be sold with a prescription from a registered medical practitioner. (*BMJ*, 4 Dec 2012)

• India's vaccine industry has been cleared by the World Health Organization (WHO) to export vaccines globally. This opens the market from major buyers and international procurement agencies, like the Gates Foundation, Clinton Foundation, UNICEF and GAVI. (*Times of India*, 15 Dec 2012)

## THE AMERICAS

• Brazilian foreign aid policy builds on very recent national experience of development based on the agriculture industry. It seeks to export the success that helped Brazil transform from a net importer to exporter of food. Its narrow policy focus on exporting recently successful domestic programmes is rapidly gaining both attention and respect among other aid donor countries. The successes of those targeted aid initiatives usually depend on identification of key elements of the Brazilian experience that can be transferred to recipient nations. (*The Guardian*, 28 Jun 2012)

• The libel action that a UK physician Andrew Wakefield, whose study on the link between MMR vaccine and autism has been retracted from *The Lancet*, filed in Texas against the *BMJ*, its editor in chief Fiona Godlee, and the investigative journalist Brian Deer following their coverage of this story, has not been upheld in the court of law. The Travis County district judge ruled that the case could not be pursued because the Texas courts had no jurisdiction over the British defendants. (*BMJ*, 6 Aug 2012)

• Mexico recently achieved a major public health milestone, after managing to enrol nearly 53 million of the previously uninsured Mexican people in public medical insurance programs. Despite periods of economic downturns that the country went through in recent years, reaching this target ensured universal health coverage in less than a decade. (*Harvard School of Public Health*, 15 Aug 2012)

• A study by the World Bank suggests that Income inequality is decreasing in Latin America, although it has been increasing in the other regions of the world. This is encouraging news for the region, whose governments often pledge to reduce the wealth gap as one of their most important targets. (*The Guardian*, 13 Nov 2012)

• It appears that Catholic schools attendees in Calgary have managed to overturn the ban on Human Papilloma Virus (HPV) vaccination in late November this year. This outcome was preceded by a four–year controversy after Ottawa and Alberta provided a grant to vaccinate schoolgirls against HPV free of costs. Catholic schools in Calgary refused to implement the policy as a group of bishops viewed the vaccination as incompatible with Catholic teachings, presuming that vaccinating against a sexually transmitted disease would encourage early sex and promiscuity. Local doctors and activists assembled the group “HPV Calgary” and campaigned to make the Calgary Catholic School Board accept HPV vaccination. (*The Globe and Mail*, 29 Nov 2012)

